# Machine Learning and Decision Support in Critical Care

**DOI:** 10.1109/JPROC.2015.2501978

**Published:** 2016-01-25

**Authors:** Alistair E. W. Johnson, Mohammad M. Ghassemi, Shamim Nemati, Katherine E. Niehaus, David A. Clifton, Gari D. Clifford

**Affiliations:** Institute for Medical Engineering & Science, Massachusetts Institute of Technology, Boston, USA; Institute for Medical Engineering & Science, Massachusetts Institute of Technology, Boston, USA; Department of Biomedical Informatics, Emory University, Atlanta, USA; Institute of Biomedical Engineering, Department of Engineering Science, University of Oxford, Oxford, UK; Institute of Biomedical Engineering, Department of Engineering Science, University of Oxford, Oxford, UK; Department of Biomedical Informatics, Emory University, Atlanta, USA; Department of Biomedical Engineering, Georgia Institute of Technology, Atlanta, USA

**Keywords:** Critical care, feature extraction, machine learning, signal processing

## Abstract

Clinical data management systems typically provide caregiver teams with useful information, derived from large, sometimes highly heterogeneous, data sources that are often changing dynamically. Over the last decade there has been a significant surge in interest in using these data sources, from simply re-using the standard clinical databases for event prediction or decision support, to including dynamic and patient-specific information into clinical monitoring and prediction problems. However, in most cases, commercial clinical databases have been designed to document clinical activity for reporting, liability and billing reasons, rather than for developing new algorithms. With increasing excitement surrounding “secondary use of medical records” and “Big Data” analytics, it is important to understand the limitations of current databases and what needs to change in order to enter an era of “precision medicine.” This review article covers many of the issues involved in the collection and preprocessing of critical care data. The three challenges in critical care are considered: compartmentalization, corruption, and complexity. A range of applications addressing these issues are covered, including the modernization of static acuity scoring; on-line patient tracking; personalized prediction and risk assessment; artifact detection; state estimation; and incorporation of multimodal data sources such as genomic and free text data.

## I. Introduction

THE intensive care unit (ICU) treats acutely ill patients in need of radical, life saving treatments. ICUs have evolved from the notion that specialized units used for close monitoring and treatment of patients could improve outcomes; many predecessors of the modern ICU were established in the late 1950s to provide respiratory support during a polio epidemic [1]. ICUs frequently have a high number of staff compared to other hospital departments, and studies have shown reduced incidence of mortality, lower hospital length of stay, and fewer illness complications [2][3], corroborating the efficacy of the intensive monitoring approach. However, real world constraints restrict the number of nurses and doctors attending to the patients in the ICU [4]. ICUs cost $81.7 billion in the US, accounting for 13.4% of hospital costs and 4.1% of national health expenditures [5]. Between 2000-2005, the number of hospital beds in the US shrank by 4.2%, but the number of critical care beds increased by 6.5% with occupancy increasing by 4.5%.

The ubiquitous monitoring of ICU patients has generated a wealth of data which presents many opportunities but also great challenges. In principle, the majority of the information required to optimally diagnose, treat and discharge a patient are present in modern ICU databases. This information is present in a plethora of formats including lab results, clinical observations, imaging scans, free text notes, genome sequences, continuous waveforms and more. The acquisition, analysis, interpretation, and presentation of this data in a clinically relevant and usable format is the premier challenge of data analysis in critical care [6].

In this review we highlight how machine learning has been used to address these challenges. In particular, we posit that data analysis in critical care faces challenges in three broad categories: compartmentalization, corruption, and complexity. Critical care data has historically been compartmentalized, with many distinct measurements of patient health being stored separately, even within the same institution. These data warehouses have been likened to silos, and the integration of data across these silos is a crucial first step before any insight can be gleaned. In the US, integrating the Medicare and Medicaid records is necessary because Medicare does not pay for nursing home services, and only by connecting these databases can costs associated with both acute and long-term care be ascertained [7]. National critical care audits have been established in many other countries including the United Kingdom, Australia, and Canada, but these databases frequently require manual entry by a skilled worker at each individual institution, rather than the automatic synchronization which is feasible with modern technology. The second challenge is the corruption of data collected during critical care. Researchers must address a multitude of sources of data corruption including sensor drop off, artifacts related to treatment interventions, and incomplete measurements. Johnson and colleagues [8] demonstrated that removal of outliers during preprocessing of data prior to development of a mortality prediction model was as important, or even more important, than the use of non-linear machine learning classifiers capable of capturing higher order interactions. Finally, and perhaps most self-evident, is the complexity inherent to critical care. ICUs provide technologically advanced life saving treatments that aim to both recover and maintain a healthy state in a very intricate and multifaceted system: the human body. The high level of monitoring in the ICU provides a unique opportunity for machine learning to provide new insights and has stimulated research into novel methods for this purpose.

**Fig 1:**
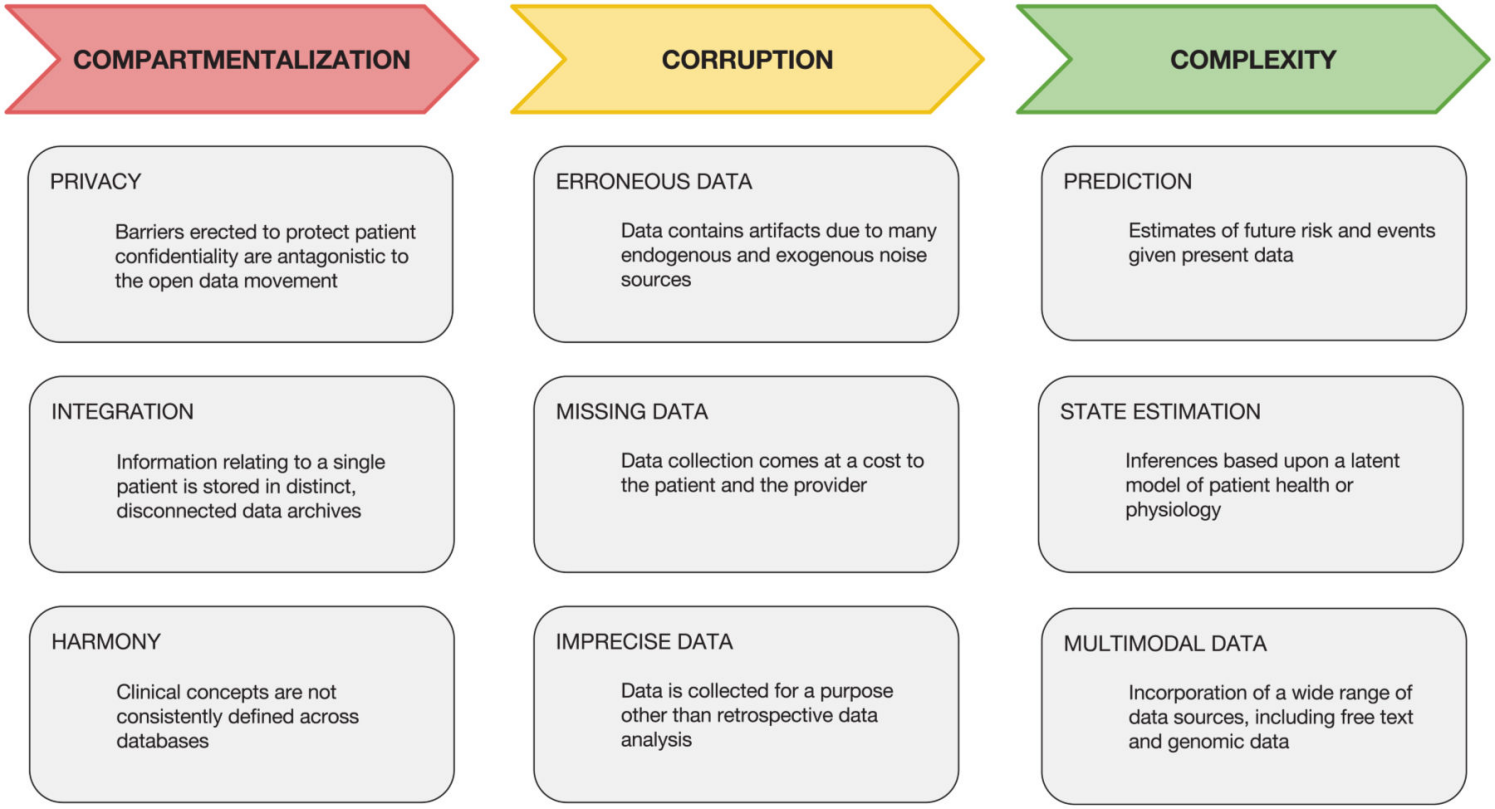
Overview of the primary challenges in critical care. The three challenges that are presented to researchers in this field are discussed in turn: the compartmentalization of the data, which results in disparate datasets that are difficult to acquire and interrogate; the corruption of the data during collection, which necessitates non-trivial corrective work; and the complexity inherent in the systems monitored.

This review provides an overview of each of these challenges and presents techniques from the field of machine learning that have been used to address them. We also discuss the future directions of research necessary to advance the field of data analytics in critical care. Figure 1 provides a diagram outlining the paper and briefly describing the topics covered. It illustrates how this article is organised along the lines of the three key challenges (the three data “C's”) in the field: *Compartmentalization*, *Corruption* and *Complexity*.

## II. Challenge 1: Compartmentalization

There are a multitude of measurements possible to quantify the current state of a patient. These measurements range from laboratory measurements performed on blood samples, real time monitoring devices quantifying vital signs, billing codes for health care visits, procedure codes for services provided within health care environments, and more. For patients admitted to the ICU, the data volume is even higher as devices continuously monitor and provide information about the patient's state. However, due to a variety of factors, all data relating to a patient's health is rarely integrated into a single system. In fact, data collected at the same institution are frequently compartmentalized. The reasons for this phenomenon are primarily as follows: the private nature of the data, the technical difficulty in integrating heterogeneous sources of data into a single location, and the challenge of harmonizing of data to facilitate its analysis.

### A. Privacy

Fundamental to the analysis of any data related to human subjects is respect of the private nature of the data. In 1996 the US Congress passed the Health Insurance Portability and Accountability Act (HIPAA) [9] which mandated confidential handling of protected health information (PHI). The National Health Service (NHS) in the United Kingdom outlined similar regulations regarding the safe keeping of PHI [10]. These acts, and their respective counterparts in different countries, are crucial for protecting the subjects of health research. While openly available computer programs and data are highly desirable to ensure the reproducibility of science [11], the private nature of the data prohibits this approach with any PHI. Data protection is achieved by health care institutions through the use of encryption protocols, access restricted systems, and strict regulations regarding the breadth and quantity of patient data which can be archived.

Inevitably, these systems have erected barriers for research using human subjects. In a survey by Ness *et al.* [12] 67.8% of respondents said that HIPAA made research more difficult (level 4 to 5 on a Likert scale), and the proportion of institutional review board applications in which the privacy rule was detrimental was significantly higher than the number of applications where the rule was beneficial.

Enabling the use of health data can be done in two formats: *restricted access* and *altered data* [13]. Restricted access entails sharing the data with a subset of approved researchers, usually at some cost and only allowing for data storage in well secured restricted locations. The second method, altered data, involves removing some aspect of the data to allow for its more general release. This could involve removing PHI from the dataset (release of data in this manner is allowed for under HIPAA safe harbor or, less frequently, the expert determination rule [14]), providing high level statistics of the data, or grouping subsets of individuals together. Selecting the optimal balance between providing useful statistical data from data and ensuring the privacy of individuals - so called “statistical disclosure control” - has been a heavily researched area [15].

Automated de-identification of free-text medical records is often the initial barrier to the analysis. Neamatullah *et al.* developed a software package which used lexical look-up tables, regular expressions, and simple heuristics to de-identify free-text medical records from PHI including doctors' names and years of dates. The investigators reported a precision and recall of 0.749 and 0.967 respectively with a fallout value of 0.002 on a test corpus [16].

The Integrating Biology and the Bedside (i2b2) project is a successful application of both methods: data is stored locally at each institution with PHI, and researchers can query for aggregate summaries of the data without access to individual level information [17]. i2b2 has also provided open access to various medical notes to encourage research in natural language processing to de-identify medical records, among other tasks. Building on this is the concept of differential privacy, where the probability of data output is almost equally likely to have been drawn from all nearly identical input data, which consequently guarantees that all outputs are insensitive to any individual's data [18]. Research has extended this concept into the unique setting of health care data and evaluated the utility of data after being anonymized using differential privacy; this may be a useful tool for future release of critical care data [19].

A notable success in the release of data in critical care is the PhysioBank component of PhysioNet [20], and in particular the Multiparameter Intelligent Monitoring in Intensive Care II (MIMIC-II) database [21], [22]. PhysioNet is a resource for openly available physiologic signals, many of which are collected during a patient's stay in critical care. MIMIC-II is a large openly available clinical database which provides de-identified patient records for over 30,000 patients admitted to the Beth Israel Deaconess Medical Center in Boston, MA, USA. The data is provided to researchers after certification of completion of a human subjects training course and the signing of a data use agreement. The database is a great step towards removing barriers between researchers and real world data necessary to validate their work. MIMIC-III has recently been released, which includes more patients and additional information regarding their individual stays (e.g. additional discharge information).

### B. Integration

There are over 200,000 medical devices registered by the U.S. Food and Drug Administration [23]. Yet there is a scarcity of interoperability among these devices. Monitoring patients in the ICU generates large volumes of data, but these data cannot be thought of as comprising one entity. Devices to measure various aspects of patient health have been developed independently and organically. One of the first treatments provided by ICUs was respiratory support [1], and ventilators, which initially only provided positive pressure through gas or pneumatic driven processes, can now electronically control volume and pressure while recording many other parameters. The ECG is one of the most frequently used measurement devices, but the data available can vary greatly: almost all devices calculate and record heart rate, but others automatically determine rhythm, ST elevation, or QT interval. Oxygen saturation devices began to be routinely used in the ICUs in the 1980s, most providing a measure of blood oxygen saturation, but some also providing heart rate. With just these few examples, it becomes clear that the integration of information from various devices into a single data management system is non trivial, requiring well defined standards for transferred packets of data, interoperability of devices, and cooperation among competitive device manufacturers. Unfortunately, there has been a lack of standardization among clinical devices [24]. The consequence of the lack of standardization and interoperability is a heterogeneous landscape of databases and record systems which can only be integrated with a great deal of labor.

The US has recently passed the Health Information Technology for Economic and Clinical Health (HITETCH) act, enforcing interoperability among various systems and partly addressing this issue. The consequences of this have been immediately apparent in the uptake of electronic health records (EHRs): in 2008 the number of US hospitals with EHRs was 9.4%, while in 2014 it had grown to 75.5% [25]. Furthermore, over 95% of these EHRs were certified, indicating that they possessed a required minimum level of interoperability. Black *et al.* [26] proposed a system for defining the quality of a database, though their concepts of coverage and accuracy do not sufficiently summarize the utility of a database, due to an equal weighting of the various components [27]. Cooke and Iwashyna [27] provide an excellent approach for selecting an existing database to address a proposed research question. The authors highlight the advantage of integrating, or linking, two datasets, providing an example where Iwashyna *et al.* [28] study quality of life among severe sepsis survivors by using an already-established link between the Health Retirement Study and Medicare files for patients admitted to ICUs. Finney *et al.* developed a data linkage scheme that allowed their hospital trust to link data from distinct databases using various identifiers with 99.8% positive predictivity [29].

Cooke and Iwashyna [27] conclude with a poignant statement - that the major barrier for optimal care for all critically ill patients is a lack of an integrated openly available data warehouse - even though this is a feasible goal. The MIMIC database has demonstrated that integration of data from disparate sources of the hospital is possible even when it requires integration of distinct databases for provider order entries, laboratory measurements, echocardiogram notes, discharge summaries, clinical observations, and mortality outcomes [21]. Furthermore, the large multi center eICU database, collected from units which take advantage of Philips Healthcare's telemetry services, has successfully integrated data from hundreds of hospitals across the continental US [30].

### C. Harmony

The integration of databases, while in itself a monumental and difficult task, provides no guarantees of a usable data set. The reason for this is the lack of data *harmony*, where a concept in one database is not linked with a concept in the other database, or the definition of concepts in one database is not congruent with the linked concept in another. An ontology is a systematic categorization of concepts, and matching ontologies is one of the largest challenges to overcome when integrating two databases. The APACHE IV mortality prediction system utilizes 114 admission diagnostic categories, and the difficulty in mapping a given ICU's diagnosis ontology to these categories has been listed as one of the major barriers to its clinical acceptance [31], [32]. Many coding schemes have been devised that aim to standardize ontologies across databases to facilitate harmonizing of their respective contents. The International Classification of Diseases (ICD) aimed to standardized all possible disease categories for patients [33], though variation in coding practice has been highlighted as a potential source of error [34]. As these codes are frequently retrospectively assigned by trained human coders reading patient notes, there is a great opportunity for natural language processing techniques to automate and improve the current work flow. The 2007 Computational Medicine Challenge provided a corpus of de-identified radiology reports and gave participants the task of assigning two codes from a set of 45 ICD-9 codes [35]. The highest performing participants used medically informed features in combination with machine learning classifiers such as C4.5. SNOMED-CT is another coding system [36] which has been shown to cover 93% of clinical concepts in a problem list [37]. Another coding system is LOINC [38], which was originally purposed for laboratory measurements but has since been extended to other clinical concepts. In fact, the growing number of distinct ontologies, many of which overlapping in purpose, has led researchers to create a database of ontologies [39]. As mentioned, the concept of interoperability has become a major area of interest due to recent US legislation changes which penalize hospitals without EHRs and stipulate requirements for their communication [25]. Yet harmony among these EHRs has yet to be achieved [40]. While other disciplines have benefited from the use of machine learning on large datasets, the lack of harmony among EHRs in critical care has stymied applications.

## III. Challenge 2: Corruption

Once data has been merged, linked, and stored in a single unified location, it is necessary to evaluate the data using some measure of quality. While preprocessing the data is a common step in many machine learning applications, it becomes critical in the medical environment because the data is collected with the intention of enhancing patient care, not to facilitate analysis. A prominent example of this phenomenon is the use of free text comments to highlight spurious readings: a high potassium measurement can be explained by a comment stating that the sample has been hemolyzed and is not an accurate reflection of the patient's health, and while this comment is trivial for a care giver to parse, it complicates retrospective analysis. Discerning true measurements from noisy observations, the hallmark of processing so called “dirty” data, is non-trivial and many pioneers in the field have created elegant solutions to these problems. Data corruption in this review has been classified into three variants: erroneous data, occurring when a value is not an accurate reflection of the true measurement; missing data, occurring when data is unavailable for a parameter of interest; and imprecise data, occurring when surrogate labels are provided instead of the desired concept label. Note that we have made a distinction between *erroneous* data, which has been modified by an aberrant phenomenon to no longer reflect the truth, and *imprecise* data, in which the data collected is accurate but does not explicitly capture the concept of interest (e.g. an ICD-9 code relating to diabetes is not identical to a diagnosis of diabetes).

### A. Erroneous data

As the removal of untrustworthy data is an important step in the training and testing of any predictive model, there is a justifiable need for algorithms that can identify artifactual data or utilize an inherent confidence measure to inform the user of questionable data. In a review by Nouira *et al.* [41], the authors note that many methods have been proposed for the task of outlier rejection in time-series analysis in the intensive care unit, including autoregression, integration, moving average (ARIMA) models [42], Bayesian forecasting [43], and a variety of robust signal estimators [44]. Three broad categories in which there can be erroneous data are explored here: waveforms, observations, and data fusion. These categories have been chosen as the type of data determines the types of artifacts possible, and consequently the various methods used to rectify the data. Waveform data continuously recorded from sensors is susceptible to high frequency artifacts associated with patient movement or clinical care. Periodic clinical measurements can be contaminated by data collection and coding practices (e.g. monitors recording missing heart rates as 0). The last category is less data specific than the previous categories, and highlights methods that take advantage of the redundant information streams in the ICU to extract data that is robust against artifacts. As these methods can be equally applied to either waveforms or observations, they have been discussed independently.

**Fig 2:**
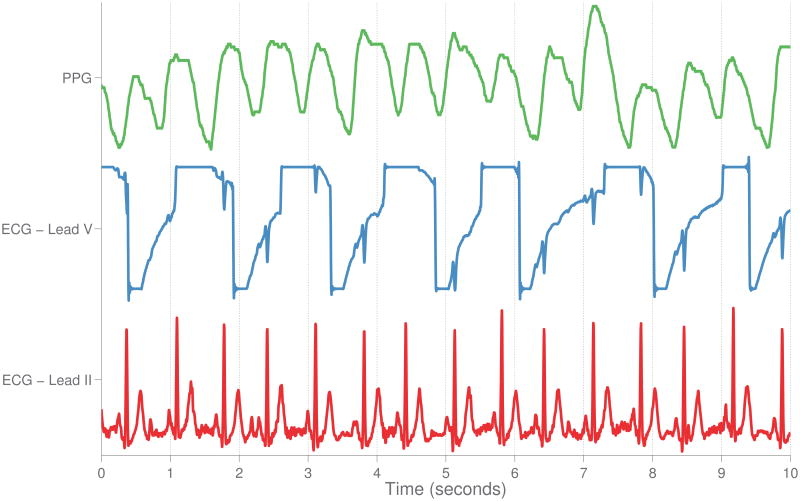
Example of a false alarm which incorrectly asserted the patient was in asystole. The signals shown are the photoplethysmogram (PPG, top in green), the electrocardiogram lead V (ECG, middle in blue), and the electrocardiogram lead II (ECG, bottom in red). The alarm likely triggered univariately on ECG lead V. At least two methods reviewed in this section could have prevented this false alarm: the use of signal quality on lead V or a multimodal data fusion approach which incorporated ECG lead II, the PPG, or both.

An example of data corruption, which resulted in a false alarm in the ICU, is given in Figure 2.

#### 1) Waveforms

A comprehensive review of artifact detection techniques in critical care is given by Nizami *et al.* [45]. The review highlights the complexity of artifact detection and removal: algorithms must be shown to generalize across units, manufacturers and varying patient demographics. Most algorithms utilize a signal quality index (SQI) which assesses how physiologically reasonable a signal is, excluding the data if it appeared invalid. Overall, the authors conclude that most existing algorithms were developed in an ad-hoc manner, lacked proper validation, were rarely evaluated in real-time, and usually not implemented in clinical practice. The authors also noted that the proprietary nature of many monitors creates an unknown element when analyzing derived signals from these monitors (e.g. unknown filters are used to process the signal prior to acquisition). This ambiguity complicates reproducibility in research and prevents algorithms developed on data acquired from one manufacturer being extended to another. Nizami *et al.* [45] also noted that a paucity of the commercially implemented signal quality indices were evaluated in the literature.

Signal quality is frequently an important quantity for real time alerting systems currently utilized in clinical practice. In a real time alerting system, the aim is to detect a sudden change in the patient state (e.g. transition from normal sinus rhythm to life threatening arrhythmia) and subsequently alert the clinical staff to this event. As discussed by Nouira *et al.* [41], these change-points are often life threatening, and ICU alarm systems were developed to alert the clinical staff with a minimal delay so as to not compromise patient care. Unfortunately, many sources of noise in the ICU are transient and imitate these change-points. This problem is further exacerbated by the simplicity of rules behind most ICU alarm systems, often utilizing simple magnitude thresholds to indicate a change of state [46], [47].

In order to evaluate the level of noise or conversely the signal quality, Li and Clifford proposed a series of techniques for pulsatile signals based on a fusion of different “simple” features [48], [49]. These features can be classified into three general categories, given their nature. The first category is based on the agreement of two independent beat detectors with different noise sensitivities. Both detectors are run simultaneously on the ECG signals, the first one being based on the detection of the ECG peak's energy [50], [51] and the second being based on the length transform [52]. Since the length transform is much more sensitive to noise than the energy detector, the level of agreement between the two detectors tends to be proportional to the level of signal quality. Other SQIs were also proposed, including features based on the power spectral density, statistical moments, and “flat line” detectors. In general, it appears that the extraction of SQIs, followed by their fusion in a machine learning framework, has had success in the literature. Behar *et al.* [53] utilized a support vector machine (SVM) [54] to directly estimate signal quality of ECG leads (achieving 95% accuracy across a variety of heart rhythms), while Li *et al.* [55] suppressed false arrhythmia alarms using SQIs and a relevance vector machine (RVM) [56] and achieved false alarm suppression rates between 17.0% for extreme bradycardia and 93.3% for asystole. Both Li *et al.* [55] and Behar *et al.* [53] highlighted the impact of rhythm type on signal quality, noting that SQIs must be tailored to a variety of arrhythmias and calling for more labelled training data to facilitate this task. More recently Morgado *et al.* [57] estimated the cross-correlation across a 12-lead ECG in combination with machine learning classifiers CART [58], C4.5 [59], RIPPER [60], and a SVM [54] to achieve an accuracy of up to 92.7% and an AUROC of up to 0.925 for the task of signal quality estimation. This method is similar to the Riemannian “potato” [61], which also uses the covariance matrix of a set of simultaneous leads to estimate signal quality. The averaging of data across time periods has also been shown to improve robustness to noise. Tsien *et al.* [62] employed decision tree induction classifiers to classify a variety of artifacts from carbon dioxide, blood pressure, heart rate and oxygen saturation trends, showing that models developed from one minute aggregations of second by second data were more accurate than those built on second by second data.

Low signal quality has a large impact on alarm systems currently in place in ICUs. Most manufacturers are conservative with alarm thresholds and tune algorithms to be extremely sensitive, resulting in a false alarm rate of up to 95% [63]. This in turn has resulted in “alarm fatigue,” which creates an unsafe patient environment due to desensitization of caregivers - life threatening events can potentially be missed. [64], [65]. Zong *et al.* [66] proposed a fuzzy logic approach to accept or reject alarms on the arterial blood pressure waveform. The algorithm maintains a running average of various physiologic measurements derived from the waveform and suppresses an alarm if one of these components is not physiologically plausible (e.g. a systolic blood pressure above 300). Additional measures of signal quality were based on comparison of the current measurements to a running average.

The recent PhysioNet / Computing in Cardiology Challenge 2015 provided a public database of 750 training and 500 test alarms to stimulate research into the area of false alarm reduction [67]. Participants in the Challenge were given samples of ICU patient waveforms that were identified by the bedside monitor as falling into one of five rhythms: asystole, extreme bradycardia, extreme tachycardia, ventricular tachycardia and ventricular fibrillation, or flutter. All submitted methods involved a form of signal quality estimation: Plesinger *et al.* [68] used physiologic thresholds on extracted features including heart rate and blood pressure, Antink *et al.* [69] used autocorrelation and a linear discriminant analysis classifier, and Fallet *et al.* [70] used mathematical morphology to provide additional robustness to noise in the underlying signal. Winning competitors were able to suppress 88% of the false alarms with a corresponding 8% true alarm suppression rate. This true alarm suppression rate dropped to 1% (with a suppression of 80% of the false alarms) when the algorithm was given an extra 30 seconds for rhythm classification. For a more detailed review of the specific issues around time-series data collection and signal processing, we would refer the reader to previous work in the literature [71].

#### 2) Observations

The framework for quality assessment and artifact removal is much more established for high resolution physiologic waveforms as compared to lower resolution clinical measurements contained in an electronic data management system (referred to here as “observations”). For such less granular information, a commonly employed technique for handling artifacts is the use of domain knowledge to remove (or disallow on input) physiologically implausible values [31], [72]. Certain measurements intrinsically lend themselves to this approach: oxygen saturation values cannot go above 100%, biochemical concentrations have known reference ranges, vital signs have implausible ranges, etc. However, the domain knowledge approach of outlier rejection has limitations. Certain variables, especially those that have logarithmic distributions, with orders of magnitude between plausible values, are not easily processed using domain knowledge. Furthermore, due to the primary use of the data for clinical care, and not retrospective modelling, these errors are often not easily corrected at the source of the data collection. Other statistical rules-of-thumb are commonly employed in place of domain knowledge (e.g. the removal of extreme percentiles, sometimes referred to as “Winsorization”) [73], [74].

**Fig 3:**
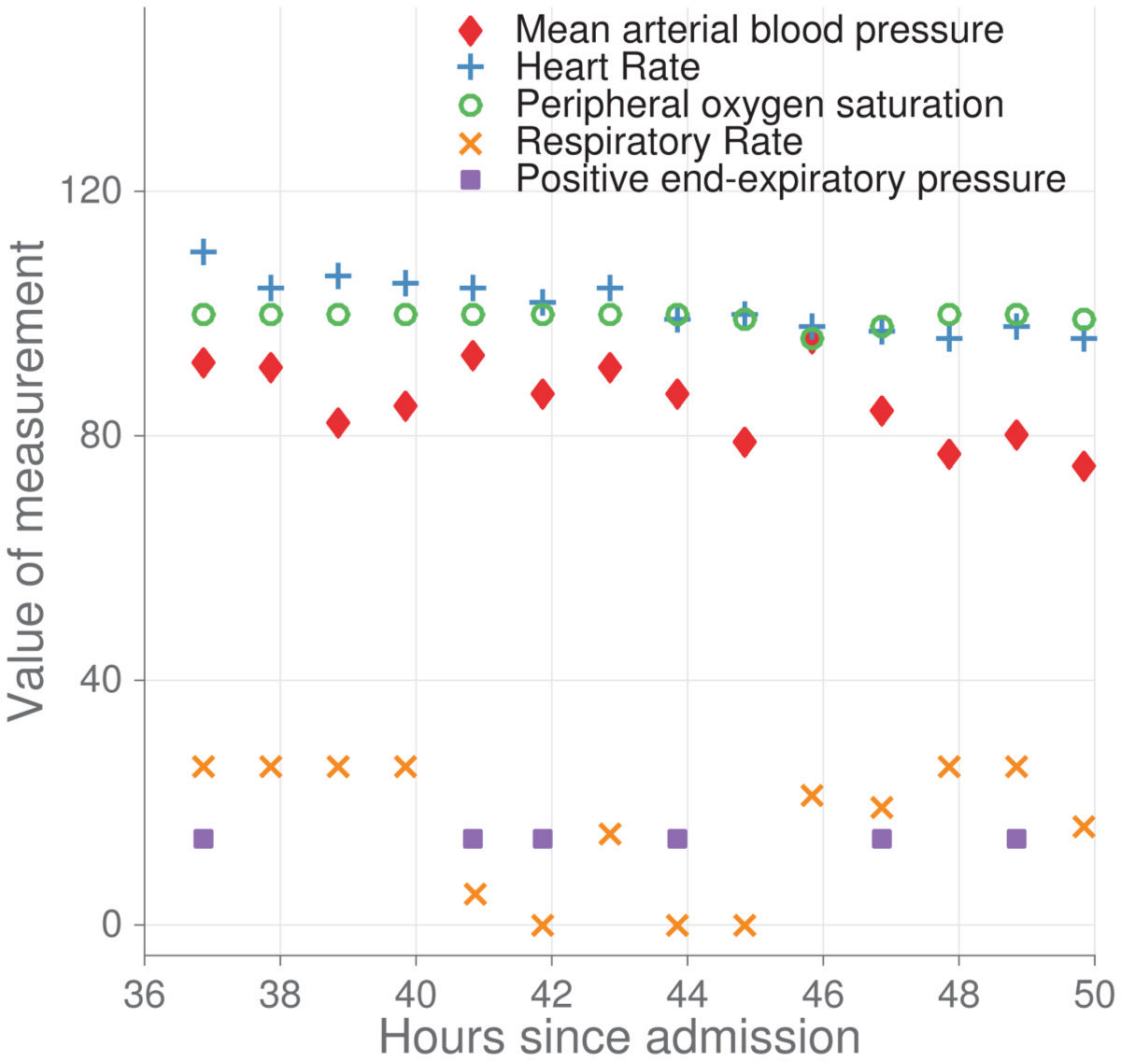
Example of low, sometimes zero respiratory rates. As a sustained breathing rate of zero for hours is incompatible with life, the data here may represent: i) undersampling of true respiratory distress with intermittent apnea, ii) erroneous data corresponding to sensor fault, or iii) manually entered data intended to represent poor physiologic state.

Fialho *et al.* [75] classified outliers as datum that were further than 1.5 times the interquartile range away from either the 25th or 75th percentile (for normally distributed data, this is approximately 2.7 standard deviations and 99.3% of the distribution resides within these limits). The authors replaced these outliers using the previous value in time, frequently referred to as sample and hold, and predicted fluid response using disease specific models. They were able to achieve AUROCs 0.04 higher than general purpose models. Johnson *et al.* demonstrated that a regularized logistic regression with no preprocessing (AUROC of 0.832) was inferior to a RF (AUROC of 0.841), but use of either domain knowledge based thresholds or an automatic method for outlier rejection resulted in the logistic regression model outperforming the RF (AUROC of 0.848 versus 0.843). They also demonstrate equivalent performance between rejection methods using automatic outliers and those relying upon domain knowledge. In their discussion of the challenge of applying knowledge-based methods, they highlight the problems of cross-institution differences in unit of measurement, labor intensity, and the lack of known thresholds for heavy tailed distributions (as noted earlier). An example of the difficulty in the identification of outliers is given in Figure 3, where the respiratory rates are implausible but may represent true respiratory distress.

Aleks *et al.* [76] considered the problem of modeling arterial-line blood pressure sensors, which are subject to frequent data artifacts and frequently cause false alarms in the ICU. They utilized a dynamic Bayesian network to model the sensor artifacts in a generative manner and reported an artifact classification performance on par with the experienced physician's. As pointed out by the authors, the problem of artifact detection is complicated by the fact that (depending on the bedside monitor brand and data archiving and streaming protocols) the sensor data are often averaged over fixed intervals, whereas the events causing data artifacts may occur at any time and often have durations significantly shorter than the data collection interval. Factorial switching linear dynamical systems (FSLDS) have been used to switch between latent modes representing stable physiology, known artifact types, and unknown noise types [77]. In particular, the authors' use of the “X-factor,” a single latent mode that captures both unknown artifact and novel physiology, gave the model additional flexibility to classify uncertain signals as abnormal, rather than forcing a decision between classifications.

Recent extensions to the FSLDS model [78] utilise a supervised framework to create a discriminative model (as opposed to a generative model) to first classify the sensor data as belonging to one of several clinical/sensor factors (e.g., blood sampling via arterial line, suction, sensor detachment, etc.) followed by inferring the underlying physiological state of the patient conditioned on each factor. This approach allows for incorporation of a richer set of features for patient state estimation and was shown to perform better for certain classes of artifact. However, the learning algorithm relies on availability of labeled data to provide a training dataset for learning various artifacts and clinical states.

Finally, we note that incorrect values are often physiologically plausible, particularly as the source monitors are designed to provide data within such ranges in the first place. Brutal filters such as sample and hold are often employed by the manufacturers (because persistence is a good estimate of physiology in the short term, and many monitors have been designed to present the best estimate “right now”). However, when using parameters derived from bedside monitors, or “clinically validated parameters,” there is a danger that significant bias and variance is introduced into the estimate, and that clinically relevant events can be missed for long periods of time. Hug *et al.* [79] demonstrated that by re-deriving blood pressures from the raw arterial blood pressure waveform, and using stringently validated signal quality indices to remove erroneous data, it is possible to see that clinical teams miss significant episodes of transient hypotension (leading to subsequent sepsis, which in turn is connected to higher mortality rates) for an average of four hours. This is an example of how, by rolling back to the original waveform data, significant extra clinical information can be extracted.

Of course, this leads to the enormous issue of labeling data (for developing quality indices and predictive algorithms). In practice, labeling of clinical data is often expensive, labor intensive, and consensus is difficult to obtain due to variations in clinical practice, inter-observer variability, human biases, and incomplete capturing of clinical context in the EHR. However, recent advances in clinical data crowd-sourcing may mitigate the problem of obtaining labeling consensus [80], [81].

As we have noted, some progress has been made in developing signal quality indices, but the vast majority of signals in the ICU lack any confidence levels. In many cases, the manufacturers of ICU medical equipment themselves generate such confidence or quality indices, but these are rarely shared (and if provided, the information is usually only displayed in the form of a traffic light system on a monitor). There is a need to open up such algorithms and require manufacturers to routinely report the confidence levels in their parameter estimates.

#### 3) Data fusion

The high level of monitoring in the ICU provides ample opportunity for methods that can fuse estimates of a given physiologic parameter from multiple sources to provide a single measurement, with high confidence in its veracity. One commonly encountered example is the estimation of heart rate, which is essential in many applications, such as the identification of extreme bradycardia or tachycardia. Such conditions frequently require immediate intervention. Since the ECG generally comprises a series of large amplitude spikes corresponding to each beat, heart rate can be estimated by event or “beat” detection algorithms [82]. Although beat detection has been well explored over the last four decades, good beat detection algorithms can still be easily confused by the high level of noise encountered in challenging recording environments. In order to increase the robustness of the heart rate extraction, fusing the estimations from different ECG channels can be highly beneficial.

Several methods have been proposed in order to improve the estimation of other physiological parameters from noisy measurements. Among the different approaches, the most obvious solutions consist in, again, aggregating the estimated values on each channel (for those parameters estimated from physiological signals collected through multiple measurement channels). For example, Jakob *et al.* [83] demonstrated that a median filter was useful for removing a large proportion (41-98%) of artifacts from blood pressure signals in post-operative cardiac patients. Yang *et. al* [84] described a technique based on an hybrid median approach where the median of a single channel is combined with median values from other channels. The resulting estimate will be accurate when no more than half the channels are corrupted, or when artifacts span less than half the width of the median window. Techniques based on signal quality assessment, a topic which has been extensively covered in the previous section, have also been successfully applied to fuse estimates of physiologic parameters from multiple signals [85], [86], [87], [88].

While the median is a robust method of fusing multiple sources of data, a variety of tractable approaches to data fusion have also been applied. The Kalman filter (KF), a state space approach, is naturally suited for the processing of time-series that frequently have artifacts [89]. KFs treat measurements, such as heart rate, as noisy observations of an underlying state (e.g. “true” heart rate), and update the state only if the confidence in the current observation is high, conditioned on the previous observation. New observations with high “innovation” are more likely to be artifacts, and these are consequently down weighted in the calculation of the state. KFs can be seen as a natural evolution of the hybrid median approach within a well defined paradigm. KFs offer the advantage of incorporating knowledge about the dynamics of the underlying signal, even in situations of great uncertainty in the observations. KF methods can identify trends and abrupt changes in the underlying (or latent) state without a large computational cost [90], [91], [92]. An approach initially proposed by Tarassenko and Townsend [93] used the KF innovation to weight heart rate derived from multiple channels. Li and Clifford [48] extended this method to include signal quality in the state updates and fusion step, thereby ensuring that low quality data and artifacts are de-weighted in the estimate of the physiological parameters.

Bayesian fusion has also recently been proposed to fuse estimates of heart rate [94], [95]. These methods treat each sensor as an independent measurement of heart rate and apply Bayes' rule to estimate the current state given the current and previous observations. Oster *et al.* [96] applied a switching KF for beat classification, allowing automatic selection of beat type from multiple “modes,” which were simultaneously evaluated. Furthermore, in a similar manner to the approach presented above [77], the method contains an extra mode unrelated to beat type, the “X-factor”, which facilitates classifying unrecognized signals as unknown. The use of an unknown class is a form of uncertainty: if the algorithm cannot be sure of a heart beat type, it is not forced to choose and can instead default to an uncertain classification. Incorporating uncertainty in medical practice has been highlighted as one of the most important components of quality improvement [97], and this should be acknowledged in models intended for use in clinical practice.

### B. Missing data

Missing data is common and difficult aspect of data collection and analysis and has been heavily researched to date [98]. Yet, clinical care infrequently acknowledges the challenges associated with the phenomenon. Vesin *et al.* [99] found that out of 44 published clinical studies, 16 did not make any mention of missing data. Worse still, only 2 out of 44 studies (less than 5%) acknowledged the importance of missing data and explicitly described the methods they addressed it with. There are three types of missing data: missing completely at random (MCAR), missing at random (MAR) and missing not at random (MNAR). Data is MCAR when the mechanism causing its absence is completely random, for example, if a laboratory machine breaks down and is unable to supply measurements for a patient. In this case, imputation of values will result in unbiased estimates. Data is MAR if the missingness mechanism is unrelated to the value of the variable. An example of data MAR would be subsequent troponin values: while an initial value may be useful in diagnosis of MI subsequent values may not be of interest and consequently would be MAR. Finally, the most difficult mechanism occurs when data is MNAR and whether the data is missing or not depends on the value of the measurement. This may be the most common mechanism of missing data as many measurements are not performed if the clinician suspects them to be normal and provide no prognostic benefit. It is worth emphasizing however that these concepts are best considered as assumptions made during an analysis, rather than properties of the data, and an analysis is not invalidated solely for making an assumption regarding the mechanism behind the missingness which may not entirely reflect reality [100].

Many methods either remove missing cases with too many missing values or impute plausible values in their place. Shah *et al.* [101] used an iterative approach incorporating singular value decomposition to impute missing data under the assumption that data were MAR. Waljee *et al.* [102] compared missing value imputation methods and demonstrated that a RF based missing value imputation method performs best in their simulation study using data which was MAR. Kim *et al.* [103] use principal component analysis in combination with EM to estimate the value of missing data from physiologic time-series.

**Fig 4:**
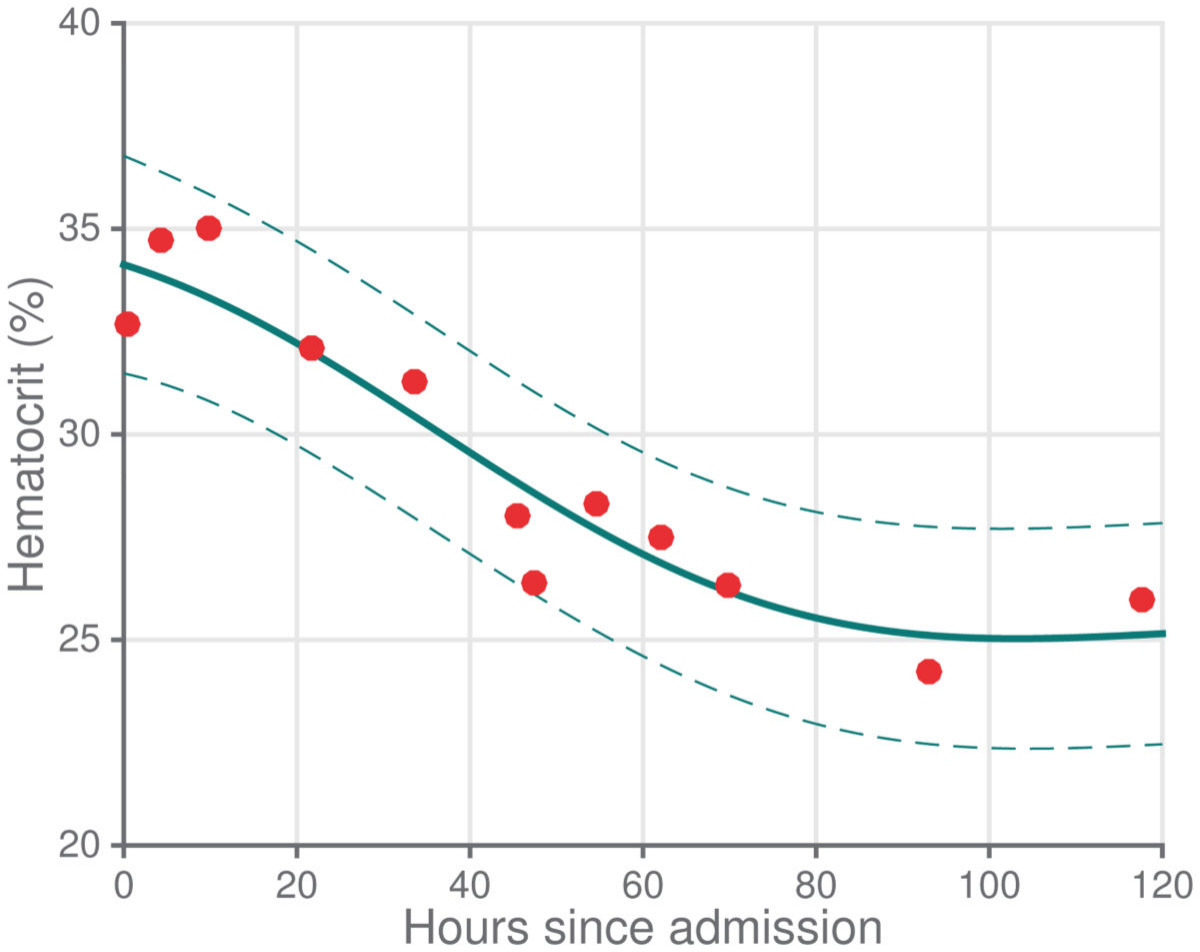
Example of a Gaussian Process (GP) regression inferring the value of missing data on an unevenly sampled time series of hematocrit values. The raw values are plotted as red circles against the mean of the GP (solid green line) and the 95% confidence intervals (dashed green lines).

Mean imputation remains one of the most common methods of missing data handling [104], and does not appear to degrade performance of various prediction systems in critical care greatly even though it assumes data is MAR [31], [72], [105]. Nevertheless, missing value imputation tends to bias the uncertainty in subsequent model estimates downward [106]. In the 1970s Dempster *et al.* [107] published an algorithm for performing Expectation-Maximization (EM) with missing data, and this represented a fundamental shift of thought among statisticians from removing missing data as a nuisance toward averaging over the uncertainty caused by missing data [106]. This paradigm shift has slowly begun to occur in critical care, though most studies have yet to acknowledge the impact of missing data [99]. Multiple imputation, a technique which involves repeatedly imputing plausible values for missing data and averaging over many instances of imputation [108], [109], has received wide praise among the medical literature but has yet to gain traction in the critical care literature [99], though this is changing [110]. Gaussian Processes (GPs) have been proposed as well as a principled method for handling missing data [111]. An example of a GP inferring data is given in Figure 4.

Lasko [112] used a nonstationary GP regression approach to explicitly estimate the time-varying volatility of latent functions to describe four laboratory values: Uric Acid (UA), Thyroid Stimulating Hormone (TSH), Creatinine (Cr), and LDL Cholesterol (LDL). Lasko estimated that these clinical laboratory tests were undersampled on average by 190% (as judged by the variables' information rate) but oversampled only by 27%. While GPs are a theoretically appealing method due to their ability to handle missing data, their use has yet to become widespread.

### C. Imprecise data

Supervised learning is a large area of machine learning that involves learning a mapping between data and an output label; learning this mapping requires training data with known labels. Unfortunately, as labels collected in critical care databases are usually recorded for purposes other than retrospective data analysis, it can be difficult to define a true “ground truth.” Frequently only surrogate annotations are available, which capture only some component of the label of interest. A further complication is the fuzzy nature of most classification tasks of interest. For example, the definition of sepsis has evolved over time, and patients who were once classified using a dichotomous diagnosis are now thought to reside within a spectrum of the disorder [113]. Even mortality, a relatively robust outcome used in many prediction tasks, is primarily used as a surrogate to quantify patient severity of illness. ICD-9 codes are frequently used to define patient diagnosis, but the use of ICD-9 codes for billing purposes has detrimentally affected the accuracy of the codes: since they are used to maximize costs, they do not necessarily best reflect patient etiology [7]. The use of ICD-9 codes as labels in supervised learning is further complicated by the fact that the codes are susceptible to coding practice changes, and patients with the same disease profile may be assigned different codes [114].

An approach used by Halpern *et al.* to derive labels from the noisy codes available in the EHR is through the use of “anchors” in place of accurate labels [115]. The authors define a feature, such as the appearance of an ICD-9 code in discharge documentation, as an anchor if and only if it is always positive when the label of interest is positive. For example, the use of insulin therapy would be an effective anchor for diabetes. A set of anchors is used to create a dataset of only positive cases, and a classifier learned using this subset of data can be generalized to apply to all positive and negative cases [116]. Through the use of a “human-in-the-loop” framework, Halpern *et al.* demonstrate that a subset of anchors can be defined which facilitate large scale unsupervised classification (since humans are required to label a subset of the data, this process is frequently referred to as semi-supervised learning).

Another common source of ground truth annotations against which an algorithm or treatment is evaluated is through manual labels provided by clinical experts. However, significant intra-and inter- observer variability and various human biases limit accuracy [117]. Even in the case of a well-described and explored field such as electrocardiography, inter-rater disagreements in ECG diagnoses and labels can be as high as 20-40% [118]. This may be due to intrinsic difficulties in interpreting the signals that are linked to the level of training or experience of the annotators [119]. Disagreements may be exacerbated by significant noise contamination due to motion artifacts, electrode contact noise, and baseline drift [120]. Moreover, the temporal window to which a label applies is often arbitrary and undefined, resulting in labels being applied to transient segments of data which fall either partially into two or more classes, or perhaps none.

Historically, inter- and intra-rater disagreements have often been ignored, and the errors associated in noisy labels have not been associated with performance measurements of classifiers. Even in cases where consensus or voting procedures have been applied, there is a risk of significant bias in the labeling. However, there have been several principled approaches which have attempted to address the issue of bias and variance in weighted voting strategies. Dawid and Skene [121] first proposed a model to probabilistically combine multiple expert annotations in an application to identify patients fit for general anaesthesia. In brief, the model learns a precision for each annotator which represents the accuracy of their annotations compared to the consensus. The estimated ground truth is calculated as a weighted sum of each annotators' label, using their precision as the weight. One of the major strengths of the approach is the ability of the EM algorithm to handle missing annotations [107]. Raykar *et al.* [122], [123] extended the algorithm to jointly model the ground truth and a regression model. Zhu *et al.* [124] demonstrated that the inclusion of contextual features, such as heart rate and signal quality, ensured that the estimated ground truth in a QT interval labelling task was always as accurate as the best human annotator without any knowledge of which annotator performed best. Welinder and Perona [125] proposed a similar model in a Bayesian framework, again estimating the precision (or inverse variance) associated with each annotator's labels. Annotator bias was incorporated into the same model for binary classification tasks by Welinder *et al.* [126]. Zhu *et al.* [127] outlined a fully Bayesian description of the model, which is capable of estimating both the precision of an annotator and their bias for continuous labels. Crowd sourcing of medical labels may be an important component in future machine learning research as it facilitates creation of large annotated databases and provides better estimates of ground truth for studies employing two or more domain experts for labeling.

## IV. Challenge 3: Complexity

Having addressed the issues around data collection and validation, the final challenge is at the core of this review: machine learning of complex data. Machine learning is simultaneously the most exciting task and the most challenging issue in critical care data analytics. The high volume of data, which frequently overwhelms care providers [128], provides ample opportunity for computerized algorithms. The research covered in this article has been grouped as follows: models that aim to predict an outcome (prediction), inferences about a latent state using measurements (state estimation), and models that analyze multiple types of data regarding a patient, including physiology or free text notes (multimodal data).

### A. Prediction

#### 1) Mortality prediction

One of the first applications of (supervised) machine learning in critical care, and indeed one of the most readily obvious applications in a unit with such severely ill patients, is the prediction of patient mortality. Prediction of patient outcomes, either time based (30 day mortality) or event based (in-hospital mortality), has been highlighted as a key component in the efficient and optimal delivery of ICU care [129]. The first model aimed at predicting severity of illness of a general ICU population was the Acute Physiology, Age, and Chronic Health Evaluation (APACHE) system [130]. The APACHE system was originally created by a panel of experts who collectively assigned higher scores for increasing physiologic abnormality. Over time, data driven analysis was incorporated into the creation of the APACHE systems to provide better models with higher performance. APACHE II simplified APACHE I by using correlation between each feature and outcome to reduce the number of features from 34 to 12 [131]. APACHE III was the first generation to utilize multivariate logistic regression to estimate the weights for each component of the model [132]. Finally, APACHE IV, the latest generation, used step-wise feature selection techniques to select a subset of covariates in the model. The steady progression of the APACHE system towards increasing reliance on data more for each subsequent generation has been echoed by other mortality prediction systems, including the Simplified Acute Severity Score (SAPS) [133], [134], [72], [105] and the Mortality Prediction Model (MPM) models [135], [136], [137]. Recent work has shown that the combination of feature selection techniques (in this case a genetic algorithm) with non-convex optimization can result in a parsimonious feature set, which provides equivalent performance to previous higher dimensional severity scores [138].

While none of the aforementioned models attained the calibration necessary to be utilized on a patient to patient basis, they have paved the way for more sophisticated machine learning methods to predict mortality and other outcomes of interest. Dybowski and colleagues [139] developed an artificial neural network (ANN) model optimized using a genetic algorithm for the purposes of mortality prediction. They demonstrated that neural networks had the flexibility to model complex patient physiology, and that this non-linear technique improved upon a logistic regression (LR) model with only linear terms. While in retrospect the study had limited power (due to the low training set size of 168 patients and large number of parameters in the neural network), it nevertheless demonstrated that the advances in machine learning could be translated into clinical practice. Clermont and colleagues later directly compared LR and ANN models [140]. When isolating the ANN's ability to model variable interactions, they showed no difference in discrimination between the LR and ANN models (AUROC of 0.848 for both). However, when allowing the ANN to directly model the relationship between the variable and the outcome, the ANN's AUROC increased to 0.857. They further demonstrated that the capability of the ANN to predict patient mortality was greatly reduced for sample sizes below 800 patients. Wong and Young similarly found a gain in discrimination from ANN models as compared to LR models (0.84 vs 0.83) [141].

The PhysioNet/Computing in Cardiology 2012 Challenge [142] aimed to stimulate research in patient specific mortality prediction systems. The primary evaluation metric, the minimum of the sensitivity (Se) and positive predictivity (PPV), was chosen to encourage algorithms to optimally classify patients who eventually died in the hospital (true positives). The best performing method, a tree based classifier with surrogate importance learned for missing data, achieved a score of 53.53%, indicating that it correctly classified half of the patients who eventually died [143]. Similar performance was achieved by set of SVMs, which were combined in a final regression step, acting as a bias correction and recalibration stage (minimum Se/PPV of 53.52%) [144]. This was a vast improvement over the (recalibrated) severity score SAPS I [133], which only achieved a score of 31.25% [142]. In a study using the openly available MIMIC-II database [20], Pirracchio and colleagues developed 12 models and an aggregate model which fused the outputs of the prior twelve (the so called “super learner”) [145]. Again, gains in performance were similar to before, with the AUROC of a regression model (0.84) increasing with the use of a more flexible model such as a random forest (0.88).

Clearly the use of regression models for prediction has been a boon for critical care, but more complicated models seem to provide little benefit in this area. One possible explanation is the exclusive use of aggregate features over large temporal windows, such as the lowest value over 24 hours. Indeed, the incorporation of features derived from patient time-series is a promising and challenging task. The concept of entropy, or the amount of disorder in the signal, can be calculated in a multitude of ways; the optimal quantification of this concept as a feature in predictive models continues to be an open area of research [146].

Saria *et al.* provide an example of how features derived from shorter-range time frames can be used in ICU prediction, in this case for preterm infants [147]. The authors used vital signs (HR, respiratory rate, and oxygen saturation) from 138 preterm infants to create a predictive risk score for severe co-morbidities. They first preprocessed the time-series data to obtain the mean and variance of both long-term and short-term trends. The resulting summary features were then modeled using long-tailed distributions, and patient log-odds ratios used to train a LR classifier to distinguish between low- and high-morbidity infants. The resulting scoring system attained an AUROC of 0.92 for predicting high morbidity, in comparison to alternative available risk scores, which had AUROCs in the range of 0.70 - 0.85.

Imhoff *et al.* [42] discuss the application of time-series analysis in the ICU for monitoring lab variables and prediction of individual patient response to therapeutic interventions, in the context of monitoring of blood pressure lactate after liver resections and acute respiratory distress syndrome.

#### 2) Medication dosing

Another important predictive question encountered in the ICU is that of medication dosing. A recent study by Ghassemi *et al.* [148] highlighted that the mis-dosing of medications in the ICU is both problematic and preventable. Their paper showed that up to two-thirds of patients at the study institution received a non-optimal initial dose of heparin and that the problem persisted regardless of the initial dose, due to the highly personal and complex factors that affect the dose-response relationship. They utilized a joint LR model and routinely collected clinical variables (e.g. race, ICU type, gender, age, and Sequential Organ Failure Assessment) to estimate a personalized initial dose of heparin. Their model had improved performance compared to a model based on weight alone (Increase in Volume Under Surface, a multi-class version of the AUC measure, of 0.06).

Ghassemi *et al.* extended their work to consider the problem of learning an optimal medication dosing policy individualized to a patent's phenotype and evolving clinical state. [149]. They describe a method for dose estimation similar to [148], but estimate optimal model parameters for each patient using a weighted combination of the incoming data from the individual and available data from a population of similar patients. They demonstrated an average improvement in AUC of 0.25, 0.19, and 0.25 for the classification of sub-therapeutic, therapeutic, and supra-therapeutic patients, respectively, and an average improvement in AUC between their personalized and a non-personalized model of greater than 0.05 for all three therapeutic states.

Recently, Nemati *et al.* proposed a deep reinforcement learning approach to sequential optimization of medications in the ICU [150]. Their technique aimed to learn latent factors in routinely collected clinical time-series, which can be directly optimized to assist in sequential adjustment of heparin dosage. They utilized a discriminative HMM for state estimation, followed by function-approximation approach to Q-learning to learn an optimal medication dosing policy. They showed that end-to-end training of the discriminative HMM and the Q-network yielded a dosing policy superior to the hospital protocol. In fact, while the expected reward over all dosing trajectories in their cohort was negative, patients whose administered heparin trajectory most closely followed the reinforcement learning agent's policy could on average expect a positive reward (that is, spending the majority of their time within the therapeutic range).

In another example, many ICU patients experience hyperglycemia in the ICU, even if not diabetic. To predict future insulin requirements, Nachimuthu, *et al.* used an expert-informed Bayesian network structure, with the values of its parameters determined using expectation maximization (to accommodate missing data) [151].

### B. State estimation

Even with the vast resources available in modern intensive care, there remain many parameters that cannot be directly measured in the ICU. For example, while many clinicians are primarily interested in evaluating cardiac output, no thoroughly validated device for its measurement is available, and various models or approximations must be utilized for its estimation. In this instance, cardiac output can be considered as a latent state, from which we measure noisy observations. In general, many aspects of patient health are not directly measurable, but can be inferred through the use of state space approaches.

#### 1) Time-series-based estimation of physiological states

Application of KFs in critical care has a long history extending beyond the artifact detection approaches discussed earlier. For instance, in the early 1980s Smith *et al.* [152] applied a KF to the time-series data from a group of kidney transplant patients, where they were able to show that in some patients, algorithmic detection of kidney rejection preceded that of experienced clinicians.

Another method for incorporating temporal information into disease prognosis is through dynamic Bayesian networks (DBNs), which are extensions of probabilistic graphical models to allow modelling of temporal data. The nodes of a DBN correspond to the random variables of interest, edges indicate the relationship between these random variables, and additional edges model the time dependency. DBNs have the desirable property that they allow for interpretation of the interactions between different variables, which is not the case for “black box” methods such as SVMs and the traditional ANNs. Gather *et al.* [153] pioneered the application of DBNs to model the conditional dependence structure of physiological variables. DBNs have been applied to the problem of parsing continuous waveforms collected at the bedside of an adult or neonatal patient for clinically significant events [154]. van der Heijden *et al.* used a DBN to model variables such as sputum volume, temperature, and blood oxygen saturation for patients with chronic obstructive pulmonary disease in order to predict exacerbation events [155].

Lehman *et al.* [169] propose an unsupervised approach for the discovery of patient state. A switching vector autoregressive (SVAR) model was applied to minute-by-minute heart rate and blood pressure measurements, with the goal of patient state estimation and clinical outcome prediction. In the absence of clinical labels for the patient time-series, an expectation-maximization algorithm was used to simultaneously segment the patient data into several phenotypic dynamical states and learn parameters of an AR model to best explain each segment. The proportion of time spent within a given dynamical region was then used as an input to a classifier for patient outcome prediction.

This approach has the advantage of automating the process of finding dynamical motifs in patient data in the absence of clinical labels, at the expense of an increase in complexity of the inference and learning algorithm. These methods have a further advantage of maintaining a belief state (that is, a probability distribution over the unobserved state variables) over the true physiological values of a patient when these cannot be directly observed due to artifact. They thus are able to provide the clinician with an estimate of the underlying true physiology, even in the presence of total corruption by noise.

#### 2) Time-series search and clustering

To enable personalized treatments, one may need to query a database for patients who match static and dynamics features of a given patient. Although much work has been performed in the field of relational database searching, the issue of searching though time-series is relatively unexplored in critical care data. Time-series search has a broad range of applications from finance to medical informatics, however, robust algorithms for finding predictive patterns in long sequences of nonstationary multivariate time-series are sparse [156]. Moreover, robust navigation and mining of physiological time-series often requires finding similar temporal patterns of physiological responses. Detection of these complex physiological patterns not only enables demarcation of important clinical events but can also elucidate hidden dynamical structures that may be suggestive of disease processes. Some specific examples where physiological signal search may be useful include real-time detection of cardiac arrhythmias, sleep staging or detection of seizure onset. In all these cases, being able to identify a cohort of patients who exhibit similar physiological dynamics could be useful in prognosis and informing treatment strategies. However, pattern recognition for physiological time-series is complicated by changes between operating regimes and measurement artifacts.

A very related topic to time-series similarity is that of time-series clustering. Clustering methods for time-series data is often more challenging than clustering of static data primarily because the distance metric between two time-series is less well-defined. Numerous distance metrics have been proposed, including the Euclidean distance, Pearson's correlation factor and dynamic time warping. As categorised by Liao, there are three different approaches for clustering time-series data: using the raw time-series as input, using features extracted from the raw data, or by presuming an underlying model of the data [157]. Unsupervised approaches can be used not only as standalone analyses, but also within two-step algorithms to generate features as input for secondary supervised analyses. This is particularly appropriate when it is unclear which aspects of the data may be discriminatory (e.g. within a complex physiologic time-series), or when it is suspected that the underlying structure in the data correlates with the desired outcome predictor variable.

Saeed *et al.* transformed patient time-series into a symbolic representation using wavelet decomposition and subsequently applied term informativeness techniques [158] to identify similar patterns in blood pressure waveforms. Lehman *et al.* [159] developed a vectorised threshold and gradient-based search engine, which allowed users to identify patients (and episodes) which fit specific criteria. By precomputing maximum values, minimum values, and gradients over multiple scales for all time-series for all patients, the authors were able to accurately identify episodes indicative of acute myocardial infarction, lactic acidosis, acute kidney injury, hemodynamic instability, multi-organ failure, and paroxysmal tachyarrhythmia. Subsequent work by the same authors [160] employed a Gaussian mixture model approach to learn the dynamic patterns in physiology through expectation maximization. Similarity between segments was computed using the Mahalanobis distance. Sow *et al.* [161] demonstrated that clustering similar patients together using locally supervised metric learning reduced the error in physiology forecasting algorithms.

In [162] authors proposed a framework for distributed identification of dynamical patterns in physiological time-series using a switching KF. Moreover, they described a fast and memory-efficient algorithm for learning and retrieval of phenotypic dynamics in large clinical time-series databases. Through simulation they showed that the proposed algorithm is at least an order of magnitude faster that the state of the art, and provided encouraging preliminary results based on real recordings of vital sign time-series from the MIMIC-II database. The switching KF framework allows for defining a notion of “similarity” among multivariate physiological time-series based on their underlying shared dynamics. Therefore, one may consider two subjects to be similar if their underlying vital sign time-series exhibit similar dynamics in response to external (e.g., tilting of body) or internal perturbations (e.g., onset of blood infection). This approach provides an improvement over time-series similarity measures based on trend-detection [163], wavelet-based symbolic representations [164], or Gaussian mixture modeling [160] due to its compact representation and sharing of the model parameters within and across time-series.

Hauskrecht *et al.* [165] applied time-series similarity measures for the opposite task: to locate abnormal patients and alert physicians when possible. The authors built a model for many possible clinical treatment actions using archived data collected in a patient's EHR. The model they developed would alert if the probability of an event, either administration of treatment or omission of treatment, strongly differed from the action taken. An example task was heparin delivery, and the model would alert if heparin was given to the current patient when the probability of heparin being given to similar patients in the past was very low. These alerts were generated using a SVM trained for each possible action, and the features were extracted from a 24 hour segmentation of patient time-series data.

[166] framed neonatal vital-signs as having an underlying set of “topics,” in an analogous manner to document clustering. This approach allowed the authors to learn the associations between different “words,” or features of the signal, and these larger “topics.” Such unsupervised analyses provided insight into patient similarities, which can drive the generation of features that are important for discrimination between patient states [147].

Schulam *et al.* [167] took a different approach to a time-series clustering model, in which they defined a set of generative linear prototype functions to describe the behaviour of individual clinical features over time for patients with scleroderma (a connective tissue disease). Ross and Dy [168] developed a set of nonparametric models for clustering patient time-series data that use a Dirichlet mixture of GPs, as well as take into account domain knowledge. In their application area of COPD patients, they were able to relate their identified subgroups to the presence of several genetic mutations known to be associated with certain forms of COPD. Though these latter two examples are drawn from applications of chronic disease, similar approaches are relevant for critical care situations.

In some applications, this two-stage procedure – unsupervised feature extraction followed by supervised learning for outcome discrimination – may be suboptimal, since the latent dynamics that are important to the supervised target may only be weakly related to those that are best for explaining the raw statistics of the time-series. Additionally, generative approaches to unsupervised feature learning [169], [170] may be hamstrung by the shortcomings of approximate inference, or the underlying models may be underspecified with respect to the nuanced features associated with the outcomes of interest. For instance, in a neurophysiological experiment involving EEG recordings, it may be the case that only a single low amplitude oscillation is the distinguishing feature of successful trials, and therefore a reduced-model specifically trained to capture that oscillation may provide a more parsimonious solution to the problem of predicting outcomes of each trial. It is therefore desirable to learn models of time-series dynamics in which the latent variables are directly tuned towards the supervised task of interest.

**Fig 5:**
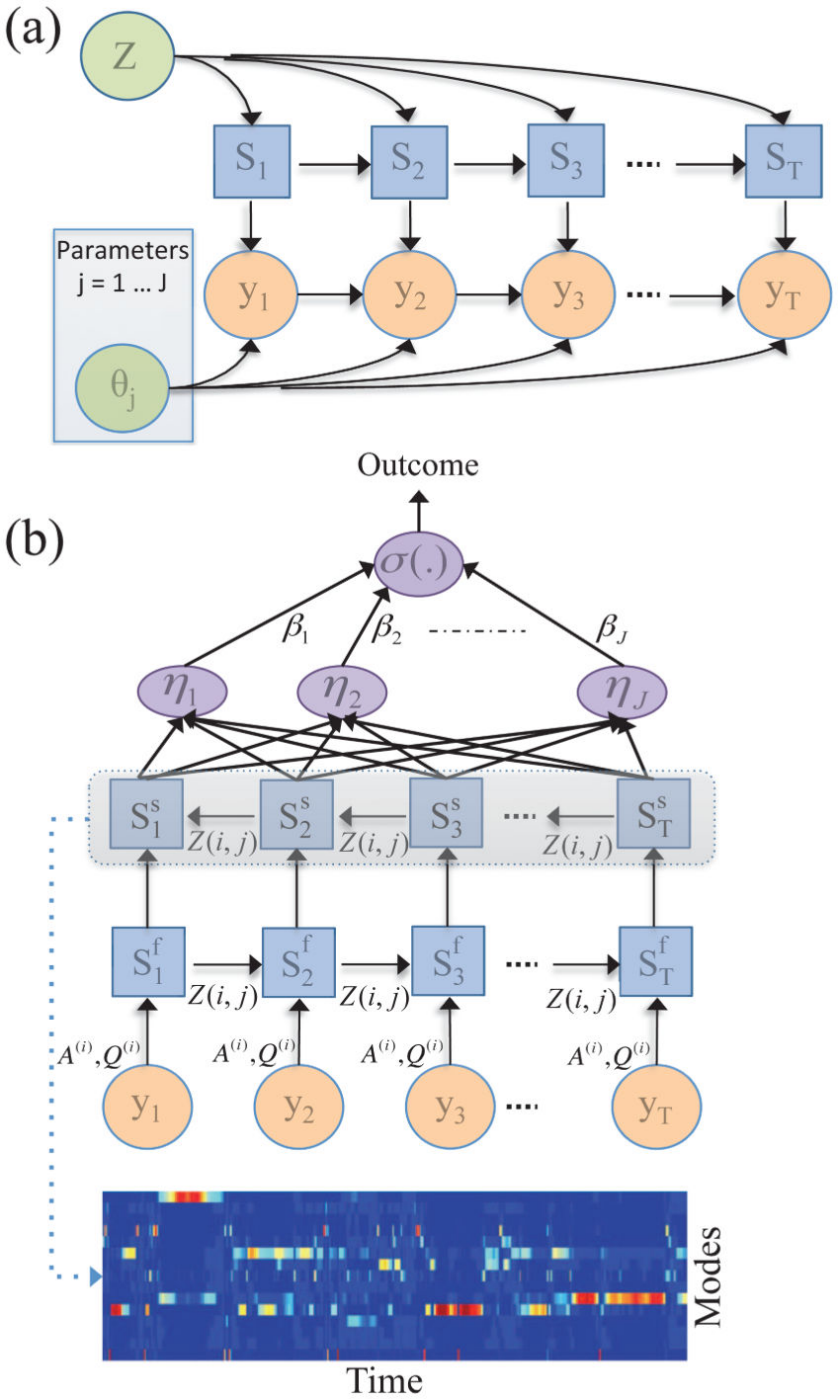
Supervised learning in dynamic Bayesian networks. Graphical model representation of the switching vector autoregressive (switching VAR) is depicted in panel (a). Panels (b) shows the unrolled representation (with respect to time and inference steps) of the two models, with an added logistic regression layer (elliptic nodes) which utilize the marginals over the discrete latent variables as features for time-series classification [an example of inferred marginals is shown at the bottom of the panel (b)]. These unrolled structures, which resemble recurrent neural networks, allow for efficient supervised learning and inference via error backpropagation.

In Nemati and Adams [171], a learning algorithm specifically designed to learn dynamical features of time-series that are directly predictive of the associated labels was presented. Rather than depending on label-free unsupervised learning to discover relevant features of the time-series, a system that expressly learns the dynamics that are most relevant for classifying time-series labels is built. The goal is to obtain compact representations of nonstationary and multivariate time-series, a task frequently referred to as (*representation learning*) [172]. To accomplish this the authors used a connection between DBNs (e.g., the switching VAR model) and ANNs to perform inference and learning in state-space models, in a manner analogous to backpropagation in neural networks [173]. This connection stems from the observation that the directed acyclic graph structure of a state-space model can be unrolled both as a function of time and inference steps to yield a deterministic neural network with efficient parameter tying across time (see Fig. 5). In contrast to generative and maximum likelihood-based approaches to feature learning in time-series, the outcome-discriminative learning framework provides the learning algorithm with the outcomes (labels) corresponding to each time-series sample (e.g., supine, slow-tilt, etc) or the entire time-series (responders vs. non-responders), and learns time-series features that are maximally discriminative. The method allowed for combining unsupervised dynamics discovery with supervised fine-tuning to design and initialize a new class of models for dynamic phenotyping, and development of phenotype-informed predictive models.

### C. Specific advances in modeling

There are some modeling advances that are worth mentioning specifically, as they are particularly useful in the face of the complexity of data found in critical care settings.

#### 1) Non-parametric Bayesian Approaches

The new field of Bayesian nonparametrics has gained much attention in recent years due to the fact that it offers a tractable means of tackling “big data” problems, where the complexity of models can scale with the increasing size and complexity of the data that are encountered.

As with conventional (parametric) Bayesian methods, non-parametric Bayesian algorithms allow the specification of prior knowledge in a principled manner, but where the distributions involved are typically defined over objects of infinite dimensionality [174]. This yields models that make fewer constraining assumptions about the underlying mechanism assumed to have generated the observed data, and which therefore offer the possibility of scaling to very large datasets that would otherwise not be possible. For example, rather than assuming that a time-series of physiological data comprises a number of individual data-points that are independent and identically-distributed (i.i.d.) with respect to some underlying probability distribution of constrained parametric form, the Bayesian nonparametric approach is to define a probability distribution over the infinite-dimensional space of functions of which the observed data are an instantiation. That is, we move from the conventional notion of point-by-point analysis, which is the current state-of-the-art in patient monitoring, to one in which entire functions are analysed (i.e., functional data analysis) [175]. This latter approach closely matches the manner in which human experts perform inference: a clinician will analyse an entire time-series by comparing it with the prior knowledge gained from their clinical training and experience, rather than by performing a series of independent decisions on each data-point within a time-series.

Clifton *et al.* illustrate how patient-specific Gaussian Process (GP) regression can be used to identify patient deterioration much earlier than would be possible using traditional methods [176]. Using wearable ECG and pulse oximetry sensors to acquire data from ambulatory patients recovering from surgery [177], the authors use GPs to model the time-series of each vital sign. A functional approach was taken in [178], and related approaches [179], [180], [181] extend extreme value statistics over highly multivariate spaces, with applications in fusing data from patient monitoring systems. Such methods were shown to perform favourably with respect to non-probabilistic systems [182].

More recent work in the area of GP-based approaches to critical care [181] demonstrated their use in combining data from wearable sensors with those obtained from manual nursing observations in acute wards. The flexibility of the GP framework was demonstrated by Durichen *et al.* [183], in which multiple time-series were fused in a Bayesian non-parametric framework for further improvements in time-series patient monitoring.

The functional approach to data analysis in critical care was used to identify common trajectories of HR and breathing rate following surgery [184]. After fitting a GP to each patient's vital signs, the authors computed a likelihood-based similarity metric between each patient-specific GP (essentially determining the likelihood that one patient's GP accurately models a second patient's time-series data). Hierarchical clustering was then used on the values of the inter-GP similarity metric to group these trajectories. Previously-unseen test data were compared to the time-series clusters to determine if the test data were similar to “normal” or “abnormal” clusters. The GP-based approach was able to more accurately discriminate normal from abnormal physiological trajectories than the state-of-the-art dynamic time warping [157]. Such techniques allow for detection of impending physiological deterioration via time-series based similarity matching of a patient to the existing patients within a database with known outcomes.

#### 2) Global optimization for cohort-specific parameter tuning

Many algorithms used for the analysis of physiological signals include hyper-parameters that must be selected by the investigator. The ultimate choice of these parameter values can have a dramatic impact on the performance of the approach [185]. Addressing this issue often requires investigators to manually tune parameters for their particular dataset. In general, global optimization approaches are best motivated for objective functions which are both costly to evaluate and whose performance is sensitive to parametrization. As concluded in Ghassemi *et al.* [186] recent advances in global optimization techniques provide an effective, and automated framework for tuning parameters of such algorithms, and easily improve upon the default settings selected by experts.

Bayesian optimization (BO) [187] is one such methodology for global optimization that relies on building and querying a relatively inexpensive probabilistic surrogate of a more expensive objective function. In general, the surrogate is a GP, which when combined with observations yields a convenient posterior distribution over functions. Intuitively, the optimization routine proceeds by exploring through seeking regions of high posterior uncertainty in the surrogate and exploiting by evaluating regions with a promising expected value. At each iteration the routine proposes a set of hyperparameters that maximizes the expected improvement over the best result seen. An experiment is run with these hyperparameters and then the surrogate model is updated with the result. This process continues over several iterations until some threshold is reached, or a maximal number of iterations surpassed.

In Ghassemi *et al.* [186] it was shown that BO can outperform the traditional global optimization techniques such as the standard grid search, multi-start scatter search algorithm, and genetic algorithms, given the same computational and time constraints.

#### 3) Growing volume of data

Many of the early studies on ICU patient prognosis relied on small samples sizes for model building, but recent trends in hardware and data collection have dramatically increased clinical database sizes. In 1981 the APACHE I system was validated on a data set of 581 admissions, while the APACHE IV system was validated in 2006 on a data set of over 44,000 patients [130], [31].

As the number of examples and feature sets grow larger, fast and efficient algorithms become more important. Fan *et al.* present an efficient method for clustering large amounts of patient data by creating a hierarchical structure [188]. Kale *et al.* present a method they term “kernalized locality-sensitive hashing” for efficiently evaluating various similarity metrics for time-series data [189].

The increasing availability of large volumes of patient data is also making it possible to apply more powerful “data hungry” machine learning techniques to clinical problems. Lasko *et al.* [190] applied a deep learning-based approach to unsupervised learning of phenotypical features in longitudinal sequences of serum uric acid measurements. The resulting unsupervised phenotypic features were passed to a classifier to distinguish the uric acid signatures of gout vs. acute leukemia, with a performance level competitive with the gold-standard features engineered by domain experts.

### D. Multimodal data

While the majority of this review has focused upon vital sign data that are commonly available in the ICU, there are many additional sources of data that can be used to improve decision support in critical care. However, care must be taken: there is not always a benefit in incorporating certain types of additional data. For instance, Saria *et al.* found that adding laboratory test values as features did not improve prediction [147], consistent with other studies that have found high amount of correlation among features [138]. The key therefore lies in appropriate combination of additional information available in the patient record.

In one novel approach, Wiens, *et al.* first created a day-by-day patient risk score for becoming infected by *Clostridium difficile* [191]. This risk score was derived from an SVM classifier with >10,000 features from the patient EHR as input. Features included the reason for admission, demographics, lab results, room location, vital sign measurements, etc (binary features were created from categorical variables, which accounts for most of the high dimensionality). The authors then modeled this risk score as a time-series, using three different approaches (extracted features, similarity metrics, and HMMs) to perform classification. Their methods were able to predict patient risk more successfully than traditional approaches of taking aggregate or daily features, with AUROCs of up to 0.79 in contrast to the traditional approaches' AUROC of 0.69.

#### 1) Incorporation of Genomic Data

One particular data type that historically has not been used widely in patient decision support is that of genomic data. While our growing understanding of patient genomics and gene expression is likely to greatly improve our ability to treat disease in the future, there are a few medical areas in which machine learning applications of genomics are already being adopted.

Clinical microbiology is one such area, which impacts closely with critical care given the high risk of infection for patients who have extended ICU stays. While *human* genetic information is not yet available in most EHR and clinical decision systems, bacterial and viral DNA analysis is more manageable (due to the smaller size of such genomes when compared with the human genome) and has already started to be incorporated into some hospital systems. Using this available information, machine learning techniques have been employed to predict bacterial and viral phenotypes from the genotype. Prediction of viral drug resistance is a pressing problem for many viruses, such as Human Immunodeficiency Virus (HIV). Both rule-based methods (e.g., ANRS, Rega, and Stanford HIVdb [192]) and machine-learning techniques (e.g., geno2pheno [193]) have been developed to improve genotypic prediction of HIV drug-susceptibility. Machine-learning methods have been found to predict more accurately the response of patients to drugs in retrospective analysis than do rule-based methods used for the same task [194].

Machine learning techniques have also been used to predict virulence profiles of clinically-relevant micro-organisms. In 2014, Laabei *et al.* used whole-genome data to predict the virulence of methicillin resistant *S. aureus* using random forests [195]. Alternative methods for bacterial resistance prediction has been attempted using LR, random forests, and set covering machines [196], [197], [198].

#### 2) Mining of Free-Text Clinical Notes

Given the explanatory power of physician notes for discounting anomalous measurements (as discussed above) and their ability to capture information not easily obtained elsewhere, there is great potential for clinical notes to improve machine learning-based prediction in the ICU setting.

Lehman *et al.* [199] used a Hierarchical Dirichlet Processes (HDP) to perform patient risk stratification by combining physiologic data and topics learned from unstructured clinical notes. The authors found that the learned topic structures significantly improved the performance of the SAPS-I algorithm for mortality prediction (from 0.72 to 0.82).

Ghassemi *et al.* [200] used a multi-step pipeline to predict ICU mortality. They first used latent dirichlet allocation (LDA) to identify common words and topics recorded in ICU patient notes. They then fit multi-task GPs to the proportion of topics observed in each note in each patient's record. Finally, as features for supervised learning to predict mortality, they used the GP hyperparameters, time-averaged topic membership, and a standard ICU-admission clinical scoring system (simplified acute physiology score: SAPS-1), finding that the combination of these features provided improved predictive performance over the clinical scoring system alone.

Ghassemi *et al.* [201] also utilized an unsupervised approach to generate vector space representations of unstructured free text notes. They investigated the evolution of clinical sentiment and language complexity with respect to several categories including: mortality, time in the hospital, age, race and gender. Their analysis identified greater positive sentiment for females, unmarried patients, and patients of African ethnicity in the ICU.

Even simple counts of textual terms and completed fields in the EHR can be informative in risk prediction. Nurses have been found to document 0.9-1.5 more optional comments and 6.1 to 10 more vital signs within the 48 hours before patient death [202].

## V. Discussion

This review has summarized the latest trends in machine learning in critical care. Focus has been given to all components necessary in this field: acquisition of data, assurance of quality, and final analysis. A large amount of effort has been invested in the processing and validation of data acquired within the ICU. Many of these methods are necessary due to the relatively unique format of data collection in the ICU. When developing algorithms in other domains, such as aircraft health monitoring or finance, researchers will specifically collect data for the purpose of analysis. However, most applications of machine learning in the ICU are *secondary*, that is, the data is collected for a purpose other than for the analysis proposed. Frequently, the data collected is acquired during routine clinical care where there are little to no incentives for acquisition of accurate data. In fact, those who record the data are frequently prevented from auditing and correcting the observations due to extreme time constraints. While advanced data management systems have the opportunity to improve clinical work flow and facilitate higher quality data collection, vendors in the health care field have produced notoriously inefficient systems which lag a great deal behind similar systems in “civilian” areas [203].

The end result is a wealth of data being collected in ICUs across the world daily going to waste [204]. Of the data that has been successfully archived and retrieved, a significant amount of effort must be employed to either transform the data into a usable form or correct a variety of artifacts present. As demonstrated in this review, a number of researchers have developed excellent techniques which address these data quality issues. These methods have allowed for further processing of the data with confidence, either for outcome prediction, state estimation, or patient alerting.

While machine learning research in critical care has provided the community with a wealth of knowledge on how patient care could be improved by the use of automated algorithms assessing patients, two criticisms arise. First, while many high performance algorithms have been proposed, there has been a paucity of evidence for the efficacy of these algorithms once implemented in ICUs.

Second, an objective analysis would imply that the sophistication of the machine learning methods applied in the critical care domain lag behind those applied in other areas. Many explanations of this could be conceived, including the earlier discussed lack of consistent and reliable data management systems in hospitals. However, we would posit that one of the biggest barriers to research has been the lack of openly available standardized datasets for the purpose of benchmarking machine learning tasks. Recent advances in image classification have been achieved in no small part due to the openly available Imagenet database which contains 456,567 images for classification as of 2014 [206]. No equivalently sized database exists for critical care. Given the complexity and heterogeneity of critical care data, and the variance in clinical practices, millions of patients are needed to identify sub-cohorts of particular disease processes and the range of applied clinical actions.

Yet, there are notable success stories surrounding open data in the past. The MIT-BIH arrhythmia database [207] galvanized manufacturers into reporting, and consequently improving, performance of their algorithms on ECG signals with arrhythmia. It was clear that, prior to the release of MIT-BIH, the lack of a well defined database for this purpose not only hindered academic progress on arrhythmia detection, but also hindered the ability of manufacturers to systematically evaluate their methods. Leaps in performance similar to those achieved after the release of MIT-BIH could be attainable in a variety of machine learning tasks after the creation of suitable standardized benchmark datasets. The need for high quality databases in critical care, with information that is complete and accurate, based upon standardized definitions of clinical disorders, interventions, and outcomes has already been recognized [208]. The creation of openly available databases such as MIMIC [22] is a key step towards this goal, and the recent announcement that a subset of the eICU database [30] will be made open to the public demonstrates that this practice is becoming more common. Future directions should strive to define and describe benchmark datasets, much like the PhysioNet/Computing in Cardiology 2012 challenge defined a benchmark dataset for mortality prediction [142]. It is worth noting that the benchmark dataset for mortality prediction resulted in state-of-the-art algorithms with over 170% higher performance than their severity score predecessors [143].

Many tasks reviewed here would benefit from benchmark datasets and, more generally, further research. A large proportion of work that addressed data corruption was ultimately used for the purpose of false alarm reduction. Drew *et al.* [65] reviewed the issue of alarm fatigue associated with false alarms and suggested alarm algorithms should focus on: using all available ECG leads and extracting at least one lead with high quality data if available, providing contextual alarms based upon multiple features (e.g. only alerting staff to pre-ventricular contractions if the patient has a prolonged QT interval), accommodating and learning from human alarm threshold adjustment, and “smart” defaults which adjust to the patient using some subset of initialization data.

Quantification of a signal into states is a principled and robust approach which has been shown to work well for both arterial blood pressure artifact detection [77] and ECG beat classification [96]. In terms of artifact detection, many known signal disruptions could be quantified in this way, including calibration artifacts, suctioning artifacts (which occur when a care provider is clearing ventilation equipment for a patient), and motion artifact. The automatic determination of artifact data would facilitate future research on the relationship between physiological dynamics and patient health. In terms of beat detection, previous research has primarily addressed ventricular ectopic beats, but many arrhythmia of interest have yet to be addressed, including atrial ectopics, asystole, atrial fibrillation, atrial flutter, bundle branch block, and so on. In general, there remains a need for openly available high performance algorithms capable of segmenting a physiologic waveform into components (e.g. segmentation of the ECG into ‘P’, ‘QRS’, and ‘T’). This could be facilitated if equipment manufacturers transmitted their confidence levels in parameter estimates. Such confidence levels could be incorporated into prediction algorithms, which could be used to greatly improve performance.

Mortality prediction models appear to have reached a plateau, with the performance of the latest generation models being fairly close to their predecessors. The primary reason for such is likely the very coarse data used in the model input, usually average values over 24 hours. The incorporation of dynamics has been shown to improve these models [169], and future research is warranted in this exciting area. Many of these models could be applied to the technically similar task of predicting readmission, where a high performing model could have many ramifications due to the large economic penalties incurred to hospitals when a patient is readmitted within 30 days.

Looking even further forward, there is an urgent need for integrative and interactive machine learning solutions, with teams of machine learning researchers and clinicians – who are directly involved in patient care and data acquisition – working in tandem to generate actionable insight and value from the increasingly large and complex critical care data [205]. The data deluge has overwhelmed many clinicians and researchers, and in the future, *smart* hospitals, which utilize machine learning approaches to provide information in a context aware manner, will be necessary [128]. Dimensionality reduction and visualization techniques are exciting areas of research which have the potential of redefining the single sensor single input monitoring approach currently applied in clinical practice. Overall, a growing body of literature [6] is pointing to the clinical utility of big data in critical care to inform prognosis and to provide early predictors of potentially life-threatening conditions in the ICU. As researchers begin to pool resources to generate large open access datasets [22], the “Unreasonable Effectiveness of Data” is beginning to take effect. However, as we note in this article, the nuances of healthcare require extreme care to be taken in the acquisition and processing of critical care data. The meaningful secondary uses of EHRs can only take place if such issues are addressed. Careful consideration of the compartmentalization, corruption, and complexity of clinical data has created a unique climate of research in critical care, which has great potential.
